# Finite element analysis of the mechanical strength of a new hip Spacer

**DOI:** 10.1186/s12891-023-06562-z

**Published:** 2023-05-30

**Authors:** Hao Ge, Hongsong Yan, Xianwang Liu, Yiwei Huang, Jianchun Zeng

**Affiliations:** 1 The First Clinical Medical School, Guangzhou University of Chinese Medicine, Jichang Road 12#, District Baiyun, Guangzhou, Guangdong China; 2 Department of Orthopaedics, The First Affiliated Hospital of Guangzhou University of Chinese Medicine, Jichang Road 16#, District 22 Baiyun, Guangzhou, 510405 Guangdong China

**Keywords:** Finite element, Periprosthetic joint infection, Bone cement, Spacer

## Abstract

**Background and objective:**

At present, the influence of the internal metallic endoskeleton of Spacer on the biomechanical strength of Spacer remains unclear. The aim of this study was to analyze the mechanical stability of a novel Spacer applying a annular skeleton that mimics the structure of trabecular bone using finite element methods.

**Metheds:**

The femur models of three healthy individuals and skeletonless Spacer, K-Spacer, and AD-Spacer were assembled to create 15 3D models. Finite element analysis was performed in an Ansys Bench2022R1. Biomechanical parameters such as stress and strain of the Spacer, internal skeleton and femur were evaluated under three loads, which were applied with the maximum force borne by the hip joint (2100 N), standing on one leg (700 N), and standing on two legs (350 N). The mechanical properties of the new hip Spacer were evaluated.

**Result:**

The stresses on the medial and lateral surfaces of the AD-Spacer and K-Spacer were smaller than the stresses in the state without skeletal support. The maximum stresses on the medial and lateral surfaces of the AD-Spacer were smaller than those of the inserted K-Spacer, and the difference gradually increased with the increase of force intensity. When the skeleton diameter was increased from 3 to 4 mm, the stresses in the medial and lateral sides of the AD-Spacer and K-Spacer necks decreased. The stress of both skeletons was concentrated at the neck, but the stress of the annular skeleton was evenly distributed on the medial and lateral sides of the skeleton. The mean stress in the proximal femur was higher in femurs with K-Spacer than in femurs with AD-Spacer.

**Conclusions:**

AD-Spacer has lower stress and higher load-bearing capacity than K-Spacer, and the advantages of AD-Spacer are more obvious under the maximum load state of hip joint.

## Introduction

Total hip arthroplasty (THA) is the ideal treatment for advanced hip disease, achieving remarkable results in relieving pain, correcting deformity, and improving joint mobility. Periprosthetic joint infection (PJI) is a catastrophic complication after prosthetic joint replacement, with an incidence of 0.4–1.44% [1, 2]. PJI means loss of joint function and the need for re-hospitalization, increasing the incidence of various complications, prolonging hospitalization, and increasing the financial burden. Currently, second-stage revision remains the standard treatment for chronic PJI and is the most commonly used treatment modality [3]. The placement of an antibiotic-containing bone cement Spacer during second-stage revision surgery combined with intermittent antibiotic therapy It allows for maximum elimination of infection and reduces the risk of recurrence of infection. Thus having the highest success rate of all treatment methods [4, 5]. Over the last two decades, antibiotic-loaded hip Spacer has become a popular surgical procedure for the treatment of hip infections, with a reported success rate of > 90% [6].

The use of Spacer containing antibiotics maintains hip stability, lower extremity length and patient mobility while the infection is eradicated [7–10] Spacer reduces fibrosis within the joint and contracture of the surrounding soft tissues, improves function and reduces pain during intervals [11–13]. Many methods and techniques have been reported for manufacturing Spacer, including manual shaping, standardized molding, and standardized prefabrication [11, 14]. However, mechanical complications occur to varying degrees with either type of Spacer. Spacer complication rates reported so far are very variable, reaching up to 73% [15]. Spacer fracture [16, 17] and implant dislocation [17, 18], among others, are frequent. Insufficient mechanical strength is the main reason for these mechanical complications. Patients requiring surgical intervention due to mechanical complications of the Spacer have a lower cure rate of infection and a poorer final clinical hip evaluation compared to patients without any mechanical complications [17]. Therefore, minimizing mechanical complications after Spacer placement is essential to optimize patient outcomes.

Today, studies on the mechanical strength of the Spacer are imperfect and controversial. Schollner et al. [19] performed in vitro mechanical tests on a gentamicin-loaded hip Spacer with a gristle pin inserted, and the mean failure load was 1.6 KN. Kummer et al. [20] studied the mechanical properties of the Spacer containing a Steinman pin with an intramedullary nail. The mechanical properties of the Spacer were studied in vitro and found that the Spacer containing the Steinman pin failed at 832 N, while the nail failed at 1275 N. Although the method of reinforcement is more controversial, the insertion of the metal skeleton resulted in a significant increase in the mechanical strength of the Spacer from in vitro experiments. the mechanical stability of reinforced hip spacers versus non-reinforced spacers was investigated in vitro by Thielen et al. [21]. The mechanical strength of the rod-reinforced Spacer was 2–3 times that of the unreinforced Spacer, and the mechanical strength of the fully dry-reinforced Spacer was 5–10 times that of the unreinforced Spacer, but the ability of the fully dry-reinforced Spacer to control infection with only 2–3 mm of antibiotic-loaded bone cement on its surface has not been demonstrated.

For this purpose, our team has developed a two-layer Spacer Antibiotic Delayed Release System (AD-Spacer) with an applied ring skeleton. Inside it can be placed antibiotic-loaded calcium sulfate. The long-term and massive release of antibiotics from calcium sulfate for the treatment of periarticular prosthetic infections has been demonstrated [22]. And an annular skeleton simulating tension and pressure trabeculae is used to ensure its instrumental strength. Spacer mechanics have been studied less frequently and mostly in vitro. The aim of this study was to investigate whether the application of the AD-Spacer has better mechanical strength and mechanics than the Spacer with conventional insertion of a kerf pin (K-Spacer) using finite element methods.

## Materials and methods

### Acquisition of geometrical models

Three healthy participants between the ages of 20 and 60 years without any history of hip trauma, hormone use, or chronic alcohol use were used in this study (Table 1). 3D modeling of the femur was performed using computed tomography images acquired with the Toshiba Aquilion CT scanner at the First Affiliated Hospital of Guangzhou University of Chinese Medicine. CT recorded in the Digital Imaging and Communications in Medicine (DICOM) format, and transferred to the MIMICS 21.0 (Materialise, Leuven, Belgium) 3D image-processing software. Mimics 21.0 is a medical image processing software which allows for the visualization of 3D models using medical images.

**Table 1:** Baseline information

Patients	Sex	Age	FL(cm)^a^	LDFA
1	M	27	42.8	92.83
2	F	43	40.6	86.75
3	F	55	39.8	87.29

Abbreviations: FL, femur length; LDFA, Lateral distal angle of femur

^a^ Femur length was defined as the distance from the center of the femoral head to the intercondylar notch

The surface errors such as spikes, intersections etc. of the femur models were corrected using Geomagic Studio 10 software (Raindrop Inc. USA). After these corrections, the 3D smooth solid model was developed and imported into SolidWorks program (Dassault Systems SolidWorks Corp., USA) in STEP format.

### Fumer bone defect modles

**Fig. 1:**
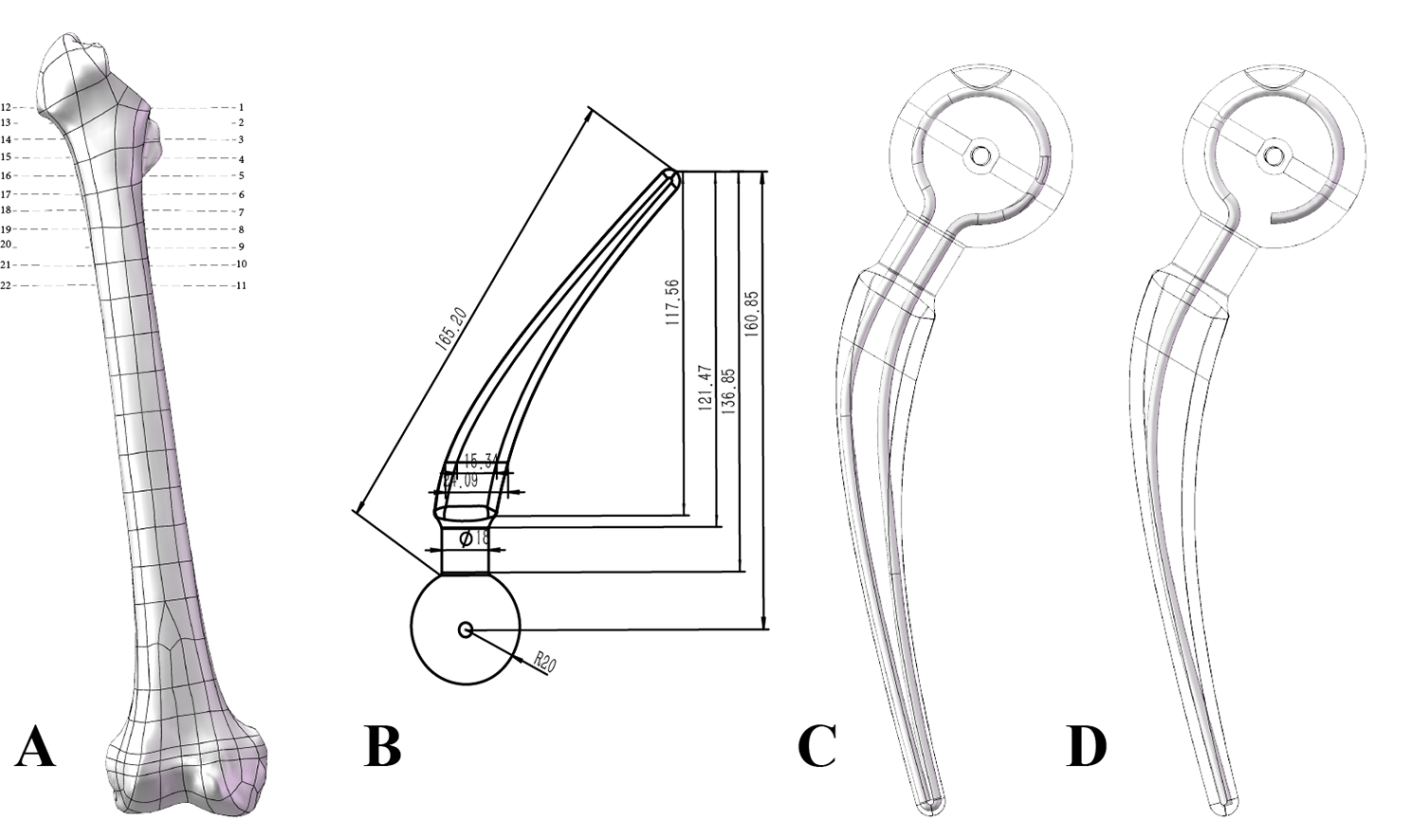
(**A**) Femur model; (**B**) Spacer model; (**C**) Annular metallic endoskeleton; (**D**) Monolayer metallic endoskeleton

The smoothed femurs were imported into SolidWorks software (2021 version, Dassault Systems SolidWorks Corp. USA) in STEP format. The head and neck of the femoral solid model were cut off 2 mm above the base of the femoral neck and perpendicular to the femoral neck. (Figure 1 A).

### Spacer modles

A Spacer housing with head diameter of 40 mm, stem length of 160 mm and neck diameter of 60 mm (Fig. 1B) was established using Solidworks software. Establishment of a traditional monolayer Kirschner wire skeleton (Fig. 1D). The annular skeleton was established by simulating the distribution of trabecular bone (Figure 1C). The diameter of the skeleton was defined in two specifications, 3 and 4 mm. The stem was 150 mm long. Then, the 3 mm AD-Spacer, 4 mm AD-Spacer, 3 mm K-Spacer and 4 mm K-Spacer models are established by Boolean operation. AD-Spacer and K-Spacer were assembled with the femur according to the surgical procedure, respectively.

### Material properties mesh and contact assignments

PMMA bone cement is a common material for Spacer. Stainless steel is a common material for Kirschner wires and is often used as the metal skeleton inside the Spacer. It was assumed that the material properties of all models were selected as linear, elastic and isotropic. According to the literature research, the material properties of cortical bone, cancellous bone, bone cement, and stainless steel were imported into Ansys workbench 2022R1(ANSYS Corporation,USA.) [23, 24]. (Table 2).

**Table 2:** The material properties of the models

	Materials	Density (kg/m3)	Modulus of elasticity (MPa)	Poison ratio
Metal skeleton	Stainless steel	7850	1.86 × 10 5	0.3
cancellous bone	cancellous bone		70	0.2
cortical bone	cortical bone		17,000	0.3
Bone cement	PMMA	1188	2500	0.35

### Mesh

**Fig. 2:**
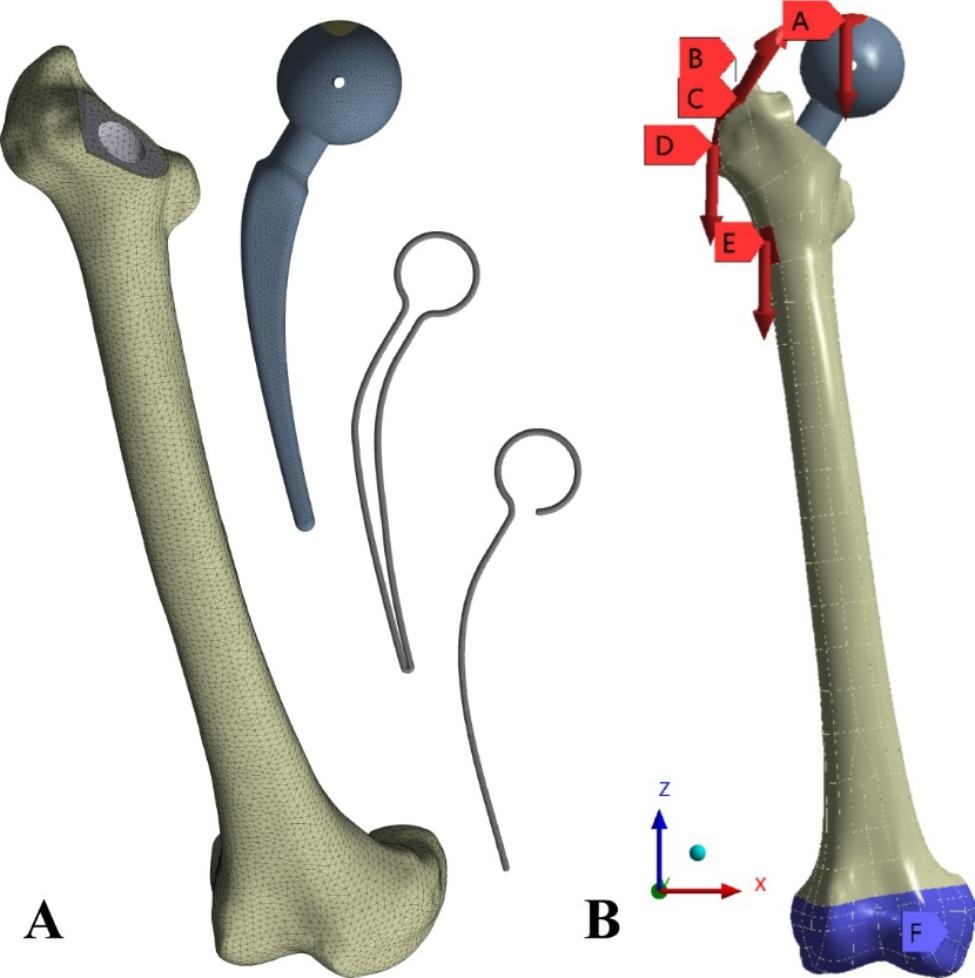
(**A**) Mesh of the femur; (**B**) Boundary conditions. A is the compressive load, B is the Abductors, C is the Vastus leteralis, D is the Tensor fascia latae lateral part, and E is the Tensor fascia latae proksimal part. F is Distal femoral restraint

The important advantage of using Solid187 tetrahedral elements throughout the FE model is its powerful ability to approximate 3D geometries, which is particularly applicable to the femur in this study [25] Convergence is obtained by means of encrypted grid. When the simulation result of the encrypted grid becomes stable, or the change amplitude of the two adjacent results is less than 5%, the result convergence is judged. On the femur model, the mesh size ranged from 6 mm to 3 mm (1 mm apart). Spacer mesh size from 6 mm to 2 mm (1 mm apart). Internal skeleton mesh size reduced from 6 mm to 1.5 mm (0.5 mm apart). Femur, Spacer, and bone reached convergence at 3, 2, and 1.5 mm meshes, respectively, as shown in Fig. 2A.

### Boundary conditions

The boundary conditions were defined for the models as seen in Fig. 2B.

Because the force on the femur is very complex, based on the restraint of the proximal femoral muscles by Duda et al. [26] (Table 3), compression loads of 350 N, 700 N, and 2000 N were applied to the femoral head, Simulated stress in standing on two legs, standing on one leg, and under maximum hip force [27, 28].

**Table 3:** Load values used in finite element analysis

Force(N)	Fx	Fy	Fz
Abductors	-406	-30.1	-605.5
Vastus leteralis	6.3	129.5	650.3
Tensor fascia latae lateral part	3.5	4.9	133
Tensor fascia latae proksimal part	-50.4	-81.2	-92.4

The boundary conditions were defined for the models as seen in Fig. 2B.

A is the compressive load, B is the Abductors, C is the Vastus leteralis, D is the Tensor fascia latae lateral part, and E is the Tensor fascia latae proksimal part. Consider the contact of the knee joint. The distal end of the femur surface was constrained with 0 degrees of freedom. (Figure 2B).

Firstly, the von Mise stress on the medial and lateral surfaces of the Spacer neck, the internal skeleton, and the medial femur was calculated to assess the risk of failure.

Second, the strains on the medial and lateral surfaces of the Spacer neck and the internal bone were calculated. Finally, the von Mises stress of the medial and lateral femur was measured at an interval of 1 cm from the lowest point of the femoral osteotomy plane (Fig. 1A) to explore the effect of Spacer implantation on the stress of the femur. The P value is less than 0.05 is considered statistically significant. All statistical analyses were performed with SPSS26.

## Results

### Model validation

The strain values of the femur under loading conditions predicted by the current FE analysis are similar to those of the experimental study, with an average difference of 6.5% between the FE calculation and the experimental results [29]. The trend of stress and strain in the proximal femur was consistent with that measured before under the same boundary conditions. The model was proved to be effective [30–32].

### Stress and strain conditions on the medial and lateral surfaces of the Spacer

**Fig. 3:**
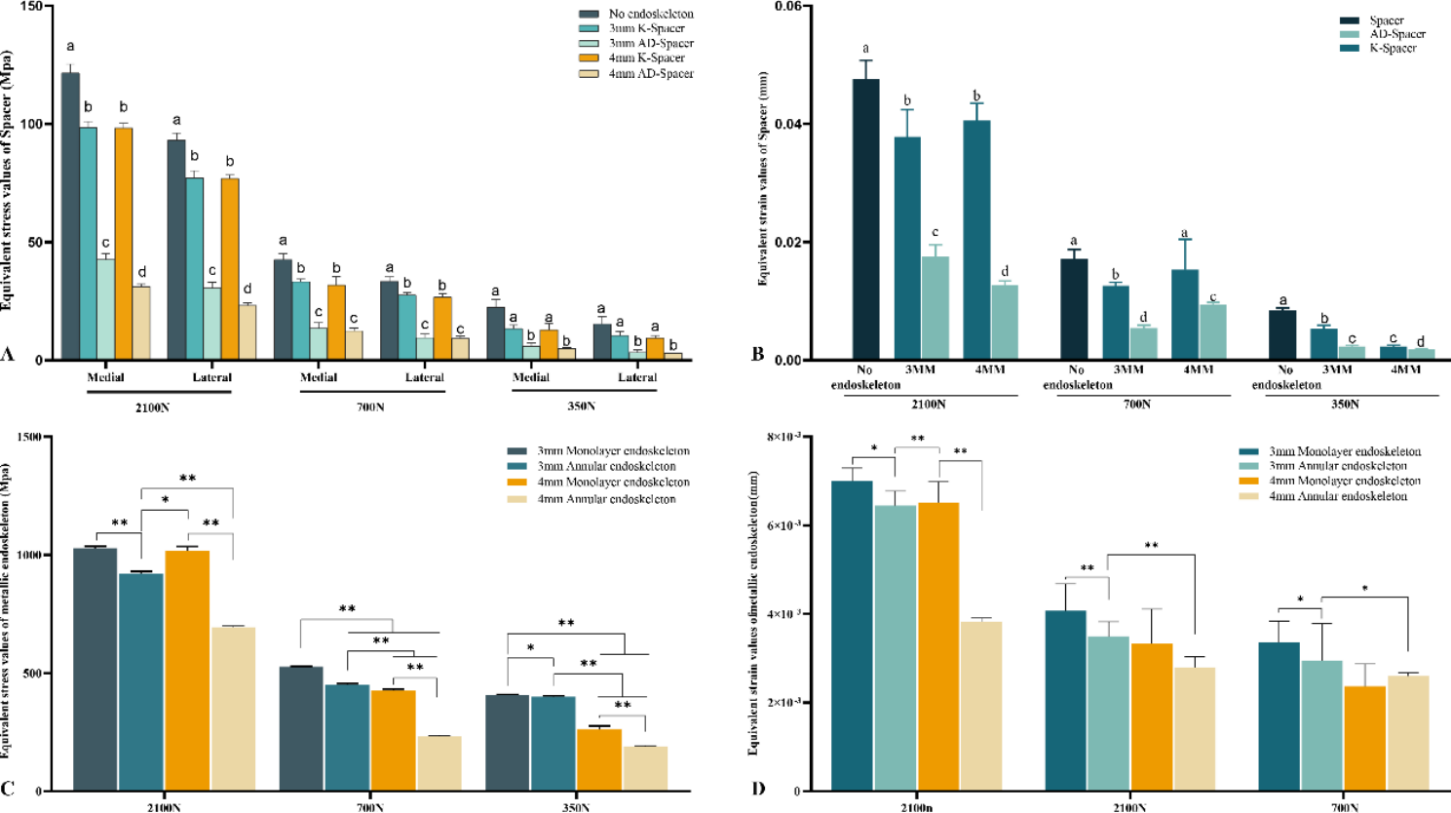
Equivalent Stress and strain values of Spacer and skeleton. (**A**) Spacer surface equivalent stress; (**B**) Spacer equivalent strain value; (**C**) Skeleton equivalent stress value; (**D**) Skeleton equivalent strain value

Figure 3A shows the maximum stress on the medial and lateral walls of the Spacer under the three loads. In this study, the medial and lateral walls stresses of AD-Spacer under three kinds of loads are significantly smaller than those of Spacer without metal skeleton support (P < 0.05).

The stress on the medial and lateral walls neck walls of the K-Spacer was significantly lower than that of the Spacer without metal skeleton support under loadings of 2100 N and 700 N, while the stress difference between the K-Spacer and the Spacer without skeleton support was not significant under loadings of 350 N.

Under the three loads, the maximum stress on the inner and outer surfaces of the AD-Spacer was significantly lower than that of the K-Spacer (P < 0.05). With the increase of force intensity, the difference gradually increased. (Fig. 3A) Under a load of 2100 N, the maximum stress difference of the medial wall of AD-Spacer and K-Spacer was 55.81 ± 1.18Mpa (3 mm skeleton) and 67.03 ± 2.99Mpa (4 mm skeleton). The stress difference of the lateral wall was 49.74 ± 0.48Mpa (3 mm skeleton) and 53.71 ± 2.42Mpa (4 mm skeleton). The maximum stress difference on the medial wall of AD-Spacer and K-Spacer under 700 N load is 19.71 ± 1.32Mpa (3 mm skeleton) and 19.32 ± 2.56 Mpa (4 mm skeleton). The average lateral difference was 18.08 ± 0.66 Mpa (3 mm skeleton) and 17.51 ± 1.69 Mpa (4 mm skeleton). Under 350 N load, the maximum stress difference of the inner wall of AD-Spacer and K-Spacer was 7.59 ± 1.66Mpa (3 mm skeleton) and 7.15 ± 1.42Mpa(4 mm skeleton), respectively. Lateral is 7.91 ± 2.56Mpa (3 mm skeleton), 6.41 ± 0.94Mpa (4 mm skeleton). Under 2100 N load, the difference of vos stress between the inner and outer walls of AD-Spacer and K-Spacer with 4 mm skeleton was the largest, which was 67.03 ± 2.99Mpa. (Table 4).

**Table 4:** Surface stress difference between K-Spacer and AD-Spacer(Mpa)

	3mm				4mm			
	Medial	Percentage	Lateral	Percentage	Medial	Percentage	Lateral	Percentage
2100N	55.81 ± 1.18	56.70%	49.74 ± 0.48	60.48%	67.03 ± 2.99	68.22%	53.706 ± 2.41	69.81%
700N	19.71 ± 1.32	59.20%	18.08 ± 0.66	65.48%	19.32 ± 2.56	60.71%	17.51 ± 1.69	65.39%
350N	7.59 ± 1.66	56.42%	7.91 ± 2.56	68.03%	7.15 ± 1.42	61.17%	6.41 ± 0.94	67.69%

As the diameter of the skeleton increased, the stress on the medial and lateral sides of the Spacer neck also decreased.

K-Spacer internal skeleton increased from 3 to 4 mm. The medial wall stress of Spacer decreases by 1.18%, 4.43% and 3.94%, respectively, under the load of 2100 N, 700 N and 350 N. The lateral wall stress decreases by 0.44%, 3.05% and 9.98% under the load of 2100 N, 700 N and 350 N, respectively. (Table 5).

**Table 5:** Surface stress difference between 3 mm skeleton Spacer and 4 mm skeleton Spacer(Mpa)

	Medial				P-vlue	Lateral				P-vlue
	K-Spacer	Percentage	AD-Spacer	Percentage		K-Spacer	Percentage	AD-Spacer	Percentage	
2100N	0.17 ± 0.78	0.18%	11.39 ± 1.53	11.60%	0.005	0.34 ± 1.55	0.44%	7.31 ± 1.67	9.50%	0.001
700N	1.47 ± 2.71	4.43%	1.08 ± 1.72	3.40%	0.877	0.84 ± 1.77	3.05%	0.38 ± 0.52	1.41%	0.674
350N	0.53 ± 2.48	3.94%	1.51 ± 0.51	11.70%	0.894	1.05 ± 0.59	9.98%	0.86 ± 0.11	9.09%	0.561

AD-Spacer internal skeleton increased from 3 to 4 mm. The medial wall stress of Spacer decreases by 11.60%, 3.40% and 11.70%, respectively, under the load of 2100 N, 700 N and 350 N. The lateral wall stress decreases by 9.50%, 1.41% and 9.09% respectively under the load of 2100 N, 700 N and 350 N. (Table 5).

Under the three loading conditions, the maximum elastic strain of AD-Spacer and K-Spacer was smaller than that of the Spacer without skeleton, while the elastic strain of AD-Spacer was smaller than that of K-Spacer (P < 0.05) (Fig. 3B).

**Fig. 4:**
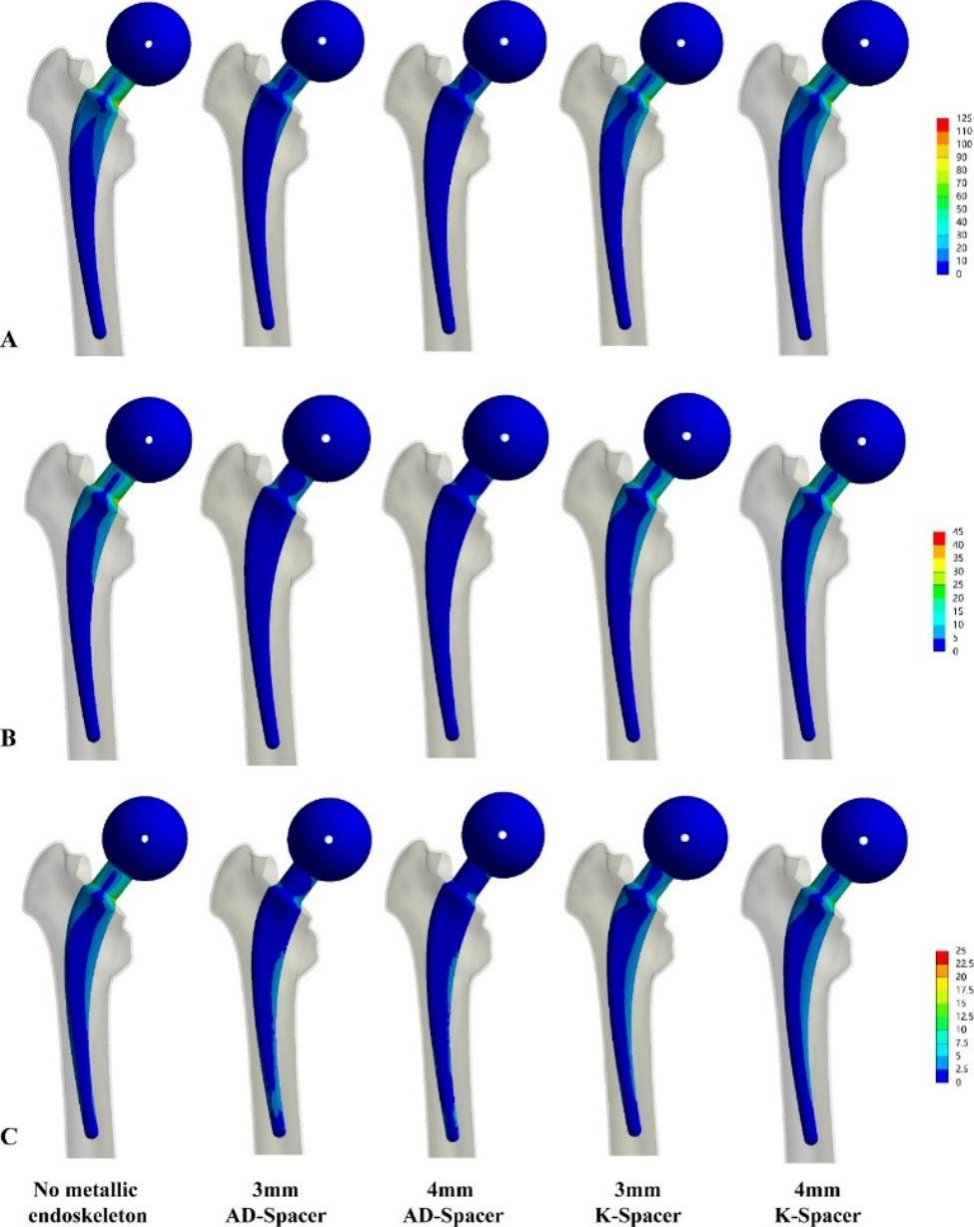
Equivalent stress values of Spacer. (**A**) Maximum force on the hip joint, 2100 N (**B**) Stand on one leg, 700 N; (**C**) Stand on two legs, 350 N

From the cloud diagram, the stresses of the AD-Space, K-Spacer and skeletonless Spacer were all concentrated in the neck, but the stress distribution of the AD-Spacer was more uniform (Fig. 4).

### Stress and strain of skeleton

**Fig. 5:**
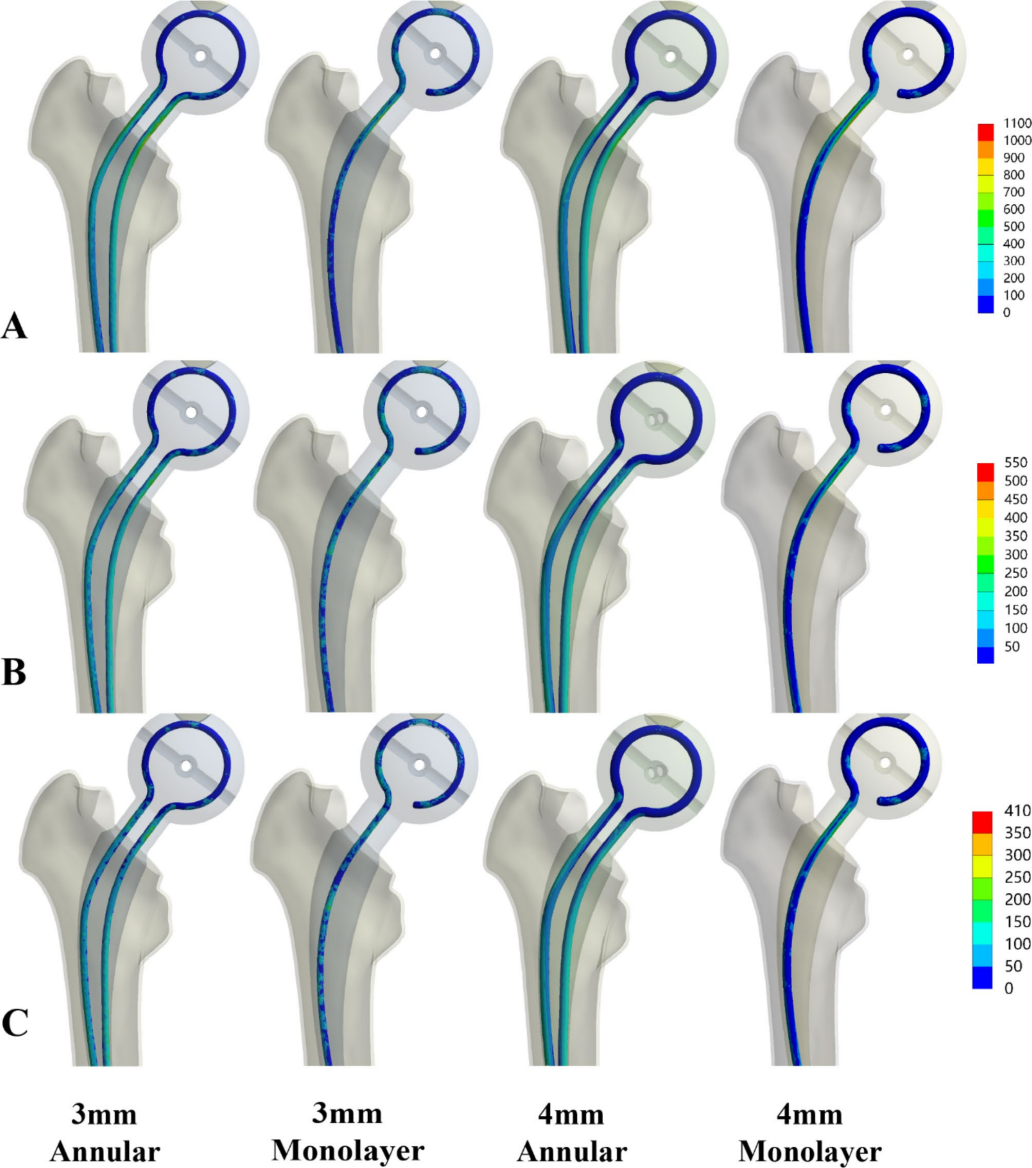
Equivalent stress values of metallic skeleton. (**A**) Maximum force on the hip joint, 2100 N (**B**) Stand on one leg, 700 N; (**C**) Stand on two legs, 350 N

The maximum stress of the internal metal skeleton is proportional to the magnitude of the force applied, and the maximum stress of the annular skeleton is smaller than that of the Kirschner wire monolayer skeleton. (Figure 3 C)The stress of both skeletons was concentrated at the neck, but the stress of the annular skeleton was evenly distributed on the medial and lateral sides of the skeleton. (Fig. 5).

Comparing the Von Mises stresses of the two endoskeletons, under the same load, the stress carried by the 3 mm endoskeletons was less than that carried by the 4 mm endoskeletons. (P < 0.05)(Figure 3 C).

### Stress of skeleton

Figure 3D shows the strain of a metal skeleton. The strain of the metal skeleton was consistent with the stress. The strain of the skeleton gradually increased with the increase of load. The strain of ring skeleton was significantly lower than that of Kirschner wire skeleton (P < 0.05). In K-Spacer, there was no significant difference in strain between 3 and 4 mm skeleton (P > 0.05). The strain of 4 mm scaffold in AD-Spacer was significantly lower than that of 3 mm scaffold (P < 0.05).

### Stress of the femur

**Fig. 6:**
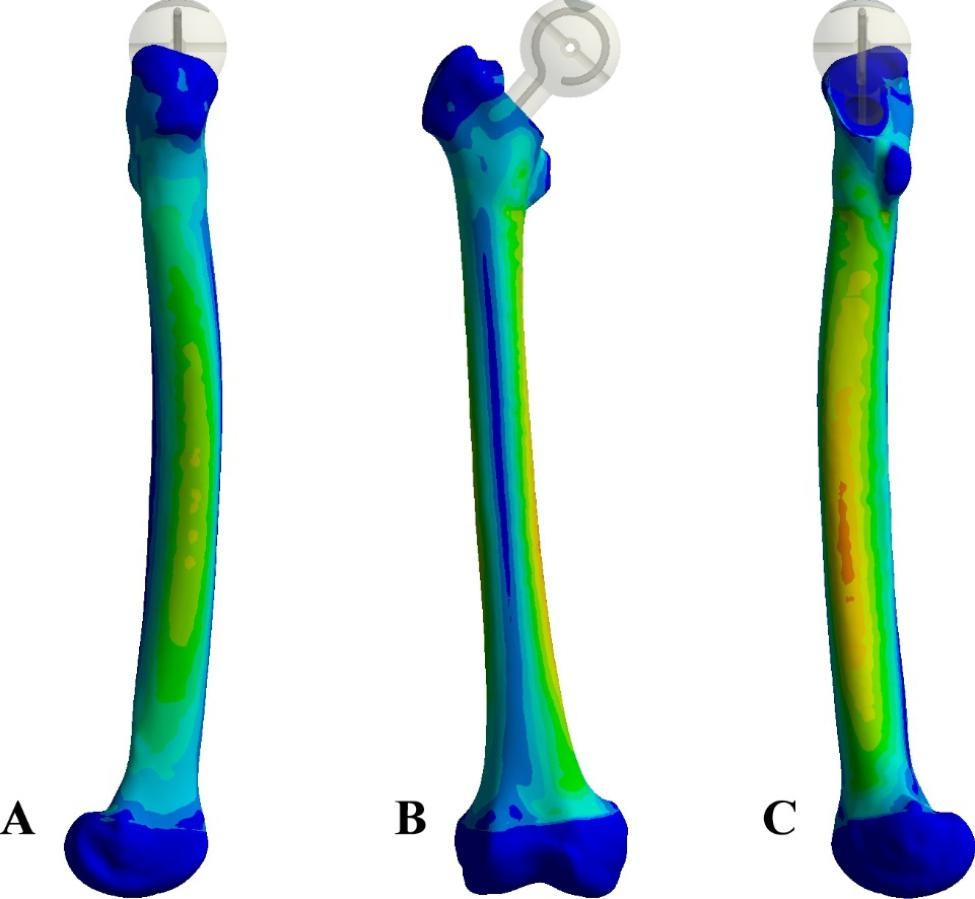
(**A**) Lateral femur; (**B**) Anterior femur; (**C**) Medial femur

The stress distribution and Von Mises stress value of the femur showed that under the action of load, the stress of the medial and lateral femurs was significantly higher than that of the anterior and posterior femurs. The stress of the medial and lateral femurs gradually increased from the proximal femur, reached the peak stress in the middle femur, and gradually decreased from the posterior to the distal femur. The maximum stress was consistent with the trend of Spacer stress. (Fig. 6). There was no significant difference in the stress distribution of the femur under the three loads.

**Fig. 7:**
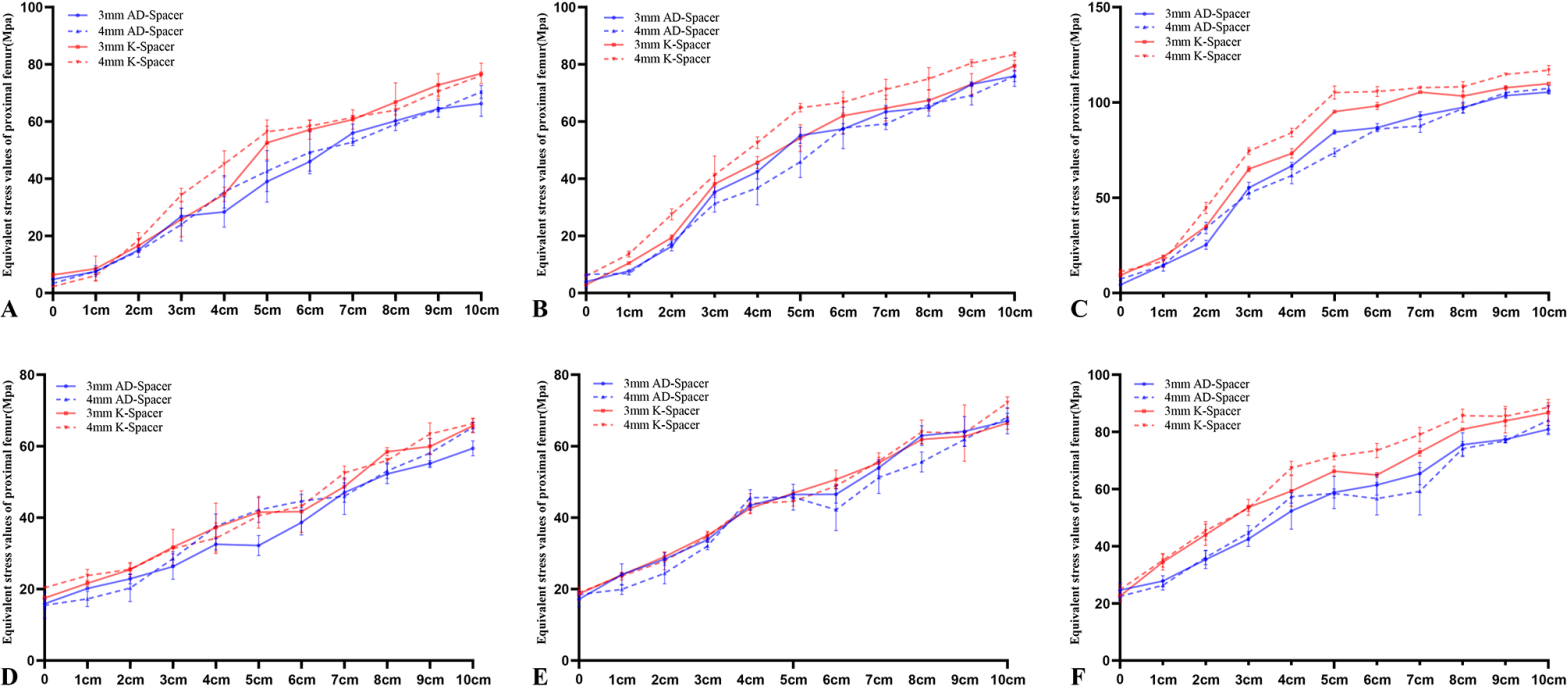
Trend of stress variation in Proximal femur. (**A**) Medial side of the proximal femur under 350 N load; (**B**) Medial side of the proximal femur under 700 N load; (**C**) Medial side of the proximal femur under 350 N load; (**D**) Lateral proximal femur under 350 N load; (**E**) Lateral proximal femur under 700 N load; (**F**) Lateral proximal femur under 2100 N load

In the proximal femur, the amount was taken from the mean stress 10 cm below the lowest point of the osteotomy plane. The mean stress in the middle and upper segments of the femur with K-Spacer was higher than that with AD-Spacer. (Fig. 7).

## Discussion

In this study, a finite element method was used to compare the mechanical strength of AD-Spacer with the application of a ring skeleton simulating the structure of bone trabeculae with that of K-Spacer with the application of a conventional single-layer kerf pin skeleton. The results show that AD-Spacer has better mechanical strength in all three stress states. The K-Spacer, on the other hand, had worse mechanical strength and failed when subjected to the maximum force on the hip joint beyond its maximum compressive strength.

In second-stage revision, implantation of the hip Spacer has the ability to bring the local level of antibiotics to a high level and thus control infection. It also has the advantage of maintaining joint motion, limiting scar tissue formation, preventing soft tissue contracture, and facilitating reimplantation [6]. And the Spacer has some weight-bearing capabilities, allowing the patient to be partially weight-bearing. However, mechanical complications resulting from the implantation of an antibiotic-loaded cemented Spacer are an important cause of revision failure.

Spacer dislocation is the most frequently reported complication. The dislocation rates reported in the literature vary widely. Jung et al. [10] reported a 17% dislocation rate in their study, while Magnan et al. [16] reported a 10% dislocation rate after implantation of a standardized hip Spacer in a small study of 10 cases. spacer geometry is an important factor in the occurrence of mechanical complications, and Leunig et al. found in their study that the head-to-neck ratio was an important factor in dislocation, with a significantly lower rate of Spacer dislocation with a high head-to-neck ratio [33] which may be related to the impingement of the Spacer neck against the acetabulum during hip motion. One study proposed that a head-neck ratio greater than 2.37 increased the size of the hip replacement safety zone. In contrast, the head-neck ratio of the Spacer in this study was 2.22. (Fig. 1B) Compared with the previously reported Spacer with a higher head-neck ratio, the odds of dislocation were lower for the same amount of bone [11].

To prevent Spacer fracture, joint surgeons may consider inserting a metal endoskeleton into the Spacer; however, there is currently little literature on this topic. Kelm et al. reported an average failure load of 20 KN for antibiotic cements that did not contain any supporting metal bone. [6]. Schöllner et al. [19] investigated in vitro the mechanical properties of a gentamicin-loaded hip joint after insertion of a gristle pin The mechanical properties of the Spacer were investigated, and stress experiments showed that the average failure load was 1.6 KN and that the insertion of the Kirschner pin did not enhance its mechanical properties, but only served to prevent the displacement of Spacer fragments. This is consistent with our results, where there was no significant difference between the stresses on the inner and outer sidewalls of the K-Spacer and the Spacer without metal skeleton when a load of 2100 N was applied (Fig. 3A). Both exceeded the maximum failure strength.

Frederic et al. performed an in vitro study comparing the mechanical properties of the hip Spacer containing a Kirschner pin, a short intramedullary nail with two tension screws, respectively [20]. The results showed that; both structures fractured at significantly lower loads. And the reason for this result is most likely the uneven force of the skeleton inside the Spacer. The medial and lateral stresses and strain of the AD-Spacer in this study were significantly lower than those of the K-Spacer and the boneless Spacer (Fig. 3A). the annular skeleton in the AD-Spacer simulated the structure of pressure and tension bone trabeculae, which acted as a pressure conductor and distributed the stresses evenly on the inner and outer sides (Fig. 5) thus improving the mechanical strength.

Spacer fracture usually occurs at the neck and leads to subsequent dislocation of the head socket. This is consistent with the findings of this study, where the maximum stresses in the Spacer under three different loads were concentrated in the neck (Fig. 4). The medial side was subjected to compressive stresses and the lateral side to tensile stresses. In the case of the inserted skeleton, the stresses are distributed according to the stiffness of the material, so that the inner skeleton bears the main load.

According to previous literature PMMA bone cement has a compressive strength of 85-100Mpa and a tensile strength of 35Mpa [34]. The results showed that the inner and outer sidewalls of the Spacer neck were less than the critical value when the Spacer without the skeleton was given a load of 350 and 700 N, and partial load bearing could be achieved. When 2100 N load was applied its inner and outer sidewall stresses exceeded the critical value and there was a risk of fracture.

The maximum stresses in the inner and outer side walls of AD-Spacer, K-Spacer and Spacer without skeleton were less than the critical values under two loads of 350 and 700 N, and it can be inferred that all three Spacers have satisfactory mechanical strength under these two loads. While under the applied load of 2100 N, the inner stresses of AD-Spacer were less than the critical value, while the inner stresses of K-Spacer had a high risk of fracture between 85-100Mpa, and the outer stresses were much greater than the critical value, which did not have reliable mechanical strength. Therefore, it can be inferred that the probability of failure of K-Spacer increases significantly when converging to the maximum stress of the hip joint, while AD-Spacer has satisfactory tensile and compressive strength.

As the diameter of the internal skeleton increased from 3 to 4 mm, the surface stress of the Spacer decreased. And the maximum stress of the skeleton also decreased, and the corresponding maximum stress of the femur increased. Therefore, increasing the diameter of the skeleton is one of the ways to improve the mechanical strength of the Spacer, but only under the load of 2100 N, the stress reduction of AD-Spacer is significantly higher than that of K-Spacer (Table 5). It can be inferred that the mechanical strength of AD-Spacer is better than that of K-Spacer under high stress conditions.

Within the bone tissue of the proximal femur there is an orderly distribution of intertwined pressure trabeculae and tension trabeculae. The tension trabeculae run from the lateral edge of the greater trochanter in an inward-superior direction and end in an arc against the upper cortex of the femoral neck inside the femoral head. The pressure trabeculae, which extend from the medial cortex of the femoral head to the femoral neck in a near vertical direction, are fan-shaped [35]. The pressure trabeculae and tension trabeculae cross and fuse with each other to provide the proximal femur with ability to bear weight and resist pressure. [36]

weight-bearing and compressive capacity. Currently, manual spacers are often used as an endoskeleton, but this approach neglects the important role of the tension trabeculae. Therefore, we believe that an endoskeleton implant that mimics pressure trabeculae and tension trabeculae can provide strong pressure support while bearing the corresponding tensile stresses. In this study, the annular skeleton simulated the role of tension and pressure trabeculae in the proximal femur, and both the inner and outer sides of the skeleton assumed some of the stresses, increasing the mechanical strength of the Spacer, in contrast to the single-layer skeleton (Fig. 5). The resulting strain is also significantly smaller than that of K-Spacer.

Analysis of the change in proximal femoral stresses showed that the proximal femur stresses were higher with the application of K-Spacer than with AD-Spacer (Fig. 7), due to the higher strength of AD-Spacer than K-Spacer, resulting in stress shielding.

The new hip spacer improves the antibiotic release time and concentration while meeting the advantages of previous hip Spacer spacers. It also has a higher head-to-neck ratio reducing the risk of dislocation due to impingement in conventional Spacer, and its internally applied annulus improves the mechanical strength of the Spacer and reduces the risk of fracture. At the same time the AD-Spacer reduces the financial burden on the patient compared to a full rod reinforced prefabricated Spacer. It provides a new Spacer selection scheme for clinicians.

However, this study also has several shortcomings: (1) the hip joint is subject to multiple muscles and ligaments pulling in the normal state, this experiment simplified force model was used for finite element analysis; (2) this experiment will femur assumed as Homogeneous, isotropic material. This is different from the actual bone material properties of non-homogeneous, anisotropic different (3) The hip joint is subjected to cyclic loading in daily life, and only a few of its several static loads mechanical strengths under static loads. Regarding the new Hip Spacer of mechanical strength of the related issues. and the incidence of complications remain to be further investigated through various experiments and clinical analysis of large samples.

## Conclusion

It was shown that the AD-Spacer with simulated bone trabecular structure had higher stress compared to the conventional K-Spacer, while the AD-Spacer with applied annular skeleton had better weight-bearing capacity. The AD-Spacer has less chance of fracture than the K-Spacer and the skeletonless Spacer. It is our future research direction to Further optimization of Spacer structure and apply the high strength material into the skeleton of AD-Spacer to achieve the normal function of patient Spacer implantation.

## Data Availability

The authors declare that all the data supporting the findings of this study are available within the article.
